# Do Preexisting Abdominal Scars Threaten Wound Healing in Abdominoplasty?

**Published:** 2010-01-18

**Authors:** Michele A. Shermak, Jessie Mallalieu, David Chang

**Affiliations:** The Johns Hopkins Medical Institutions, Division of Plastic Surgery and Department of Surgery, Baltimore, Md

## Abstract

**Purpose:** Abdominal scars may impair healing after abdominoplasty. We aimed to determine whether right subcostal or upper midline scars led to increased wound healing problems. **Methods and Materials:** Review of all patients who had abdominoplasty from March 1998 to February 2008 was performed. Variables studied included age, gender, body mass index (BMI), medical history, and postoperative complications. Statistical analysis was performed in Stata SE, version 10. **Results:** Of 420 abdominoplasty procedures, 62.2% had open gastric bypass surgery (GBS) and 19% had laparoscopic GBS. Seven percent (*n* = 29) of the series had a right subcostal scar. Overall risk of any complication was 32.9%, with 18.3% risk of wound healing problem (18.3%) and seroma (14.9%). χ^2^analysis revealed a significant relationship between any abdominal scar and any complication (*P* = .001), and wound healing problem specifically (*P* = .009). The subcostal scar was significantly associated with wound healing problems (*P* = .003). The upper midline scar was not associated with wound healing or seroma complication. While multivariate analysis erased any significant relationship between abdominal scars and complications, elevated BMI presented a significant threat to wound healing. With every unit increase in BMI, a 5% increase in the risk of any complication and a 6% increased risk in wound healing was calculated (*P* = .001). There was no difference in complications between the open and laparoscopic GBS groups, indicating that the upper midline incision did not pose a threat to wound healing. **Conclusions:** Elevated BMI poses a greater threat to healing than does abdominal scar. Caution is recommended in undermining when the right subcostal scar exists.

Abdominoplasty is the third most popular surgical procedure performed in plastic surgery and is rising in prevalence, partly because of the growing number of massive weight loss (MWL) patients.^1^ Preexisting abdominal scars, particularly the right subcostal scar utilized for open cholecystectomy, commonly known as a *Kocher scar*, may threaten healing after abdominoplasty due to past sectioning and scarring of the superior blood supply necessary for abdominal healing. Abdominal scarring is prevalent in the MWL population. Many MWL patients have undergone open Roux-en-Y gastric bypass surgery (GBS), most often through an upper midline scar. Whereas the presence of abdominal scars may impact the surgical approach chosen for abdominoplasty, some surgeons believe that concerns about wound healing with right subcostal scars are overstated, particularly if the surgery was in the remote past.

In our experience, we seemed to experience more wound healing problems with subcostal scars (Fig 1). We aimed to determine whether patients with right subcostal or upper midline scars experienced increased risk of complications, and specifically, wound healing problems, after abdominoplasty procedures.

## PATIENTS AND METHODS

Review of operative and clinic notes of all patients who had abdominoplasty from March 1998 to February 2008 was performed. Variables studied included age, gender, body mass index (BMI), medical and surgical history (particularly focusing on abdominal scars), and postoperative complications. Complications included wound healing problems and seromas, as well as postoperative bleeding and venous thromboembolism. Statistical analysis was performed in Stata MP, version 10. *P* value less than or equal to .05 was considered to be significant.

## RESULTS

A total of 420 abdominoplasty procedures were performed. Within the overall group, 62.2% had open GBS with an upper midline incision and 19% had laparoscopic GBS (Table 1). Seven percent (*n* = 29) of the patients had a Kocher scar. Mean BMI at the time of abdominoplasty was 33 (range: 19.5–88).

Overall risk of any complication was 32.9%, with risk of wound healing problem (18.3%) and seroma (14.9%). χ^2^ analysis revealed significance in the relationship between any abdominal scar and any complication (*P* = .001), and wound healing problem as a specific complication (*P* = .009) (Tables 2 and 3). The Kocher scar was significantly associated with wound healing problems (*P* = .003) but not with seromas (Table 4). The upper midline incision also had a higher association with any complication but not with wound healing or seroma complication.

Multivariate analysis, controlling for age, gender, medical comorbidities, smoking history, and BMI, indicated that no significant relationship existed between abdominal scars and postoperative complications. While this relationship dropped out in the adjusted analysis, BMI revealed itself to be the variable providing the greatest detriment to successful wound healing and uncomplicated recovery (Tables 5 and 6). With every unit increase in BMI, there was a 5% increase in the risk of any complication and a 6% increased risk in wound healing (*P* = .001). Female gender provided a positive benefit in wound healing (*P* = .006; Table 5). There was no difference in complications between the open and the laparoscopic GBS groups, indicating that the upper midline incision did not pose a threat to wound healing.

## DISCUSSION

While the Kocher scar seems to pose a threat to wound healing in abdominoplasty, multivariate analysis controlling for a range of variables reveals that it is BMI, not the Kocher scar, that poses the greater threat. Upper midline incisions also do not present a risk to wound healing in abdominoplasty.

The breast reconstruction literature has investigated the impact of abdominal scars on outcomes in procedures utilizing autogenous abdominal tissue, such as free TRAM flaps and DIEP flaps. Concerns are related not only to abdominal healing after flap harvest but also on flap healing. Parrett et al^2^ retrospectively analyzed a 3-year series of 168 DIEP flaps, 78 with a range of prior abdominal scars. Although they found that flap outcome was unaffected, abdominal donor site healing was notably impaired, including abdominal wound breakdown, seroma, and bulges of the abdominal wall.^2^ Losken et al^3^ studied the impact of right subcostal scars on breast and donor site morbidity with TRAM flap surgery and found that subcostal scars paired with smoking led to a 6.5-fold increase in the risk of abdominal wall necrosis.^3^ These papers as well as one by Schoeller et al^4^ submit recommendations to avert problems, including minimized undermining and modification of incisions utilized to optimize circulation to the healing abdominal incision.^2^^-^4

Although caution is recommended in undermining when a Kocher scar exists, we found that including BMI in the analysis disabled the risk of Kocher scar in causing wound healing problems. This makes sense considering the fact that subcostal scars are more common in more obese patients. BMI has been identified in many studies as a negative predictor of wound healing in abdominal surgery and in breast reconstruction surgery.^5^^-^10

We also found in this analysis that women fared better than men in abdominoplasty outcome. We have demonstrated in the past that male gender proved to be detrimental to wound healing in postbariatric body contouring surgery.^5^ Whether this occurs secondary to the beneficial effects of estrogen, from greater compliance on the part of the female patient population, or for other reasons, this study supports the finding that female patients experience better outcomes than male patients.

**Figure 2:**
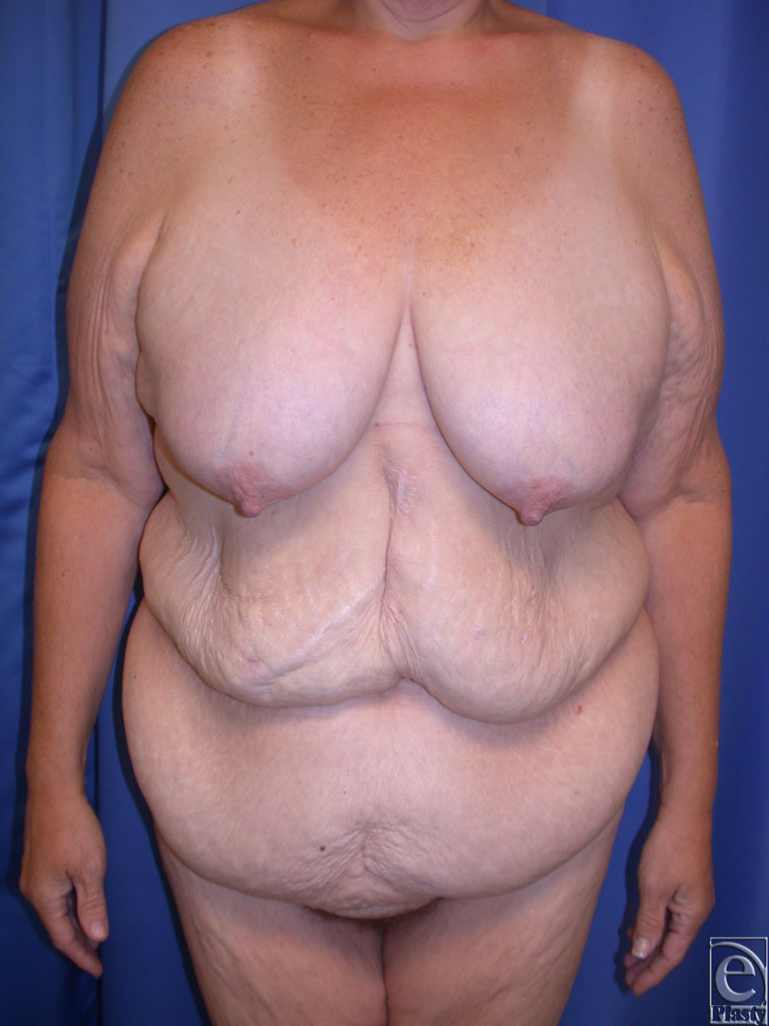

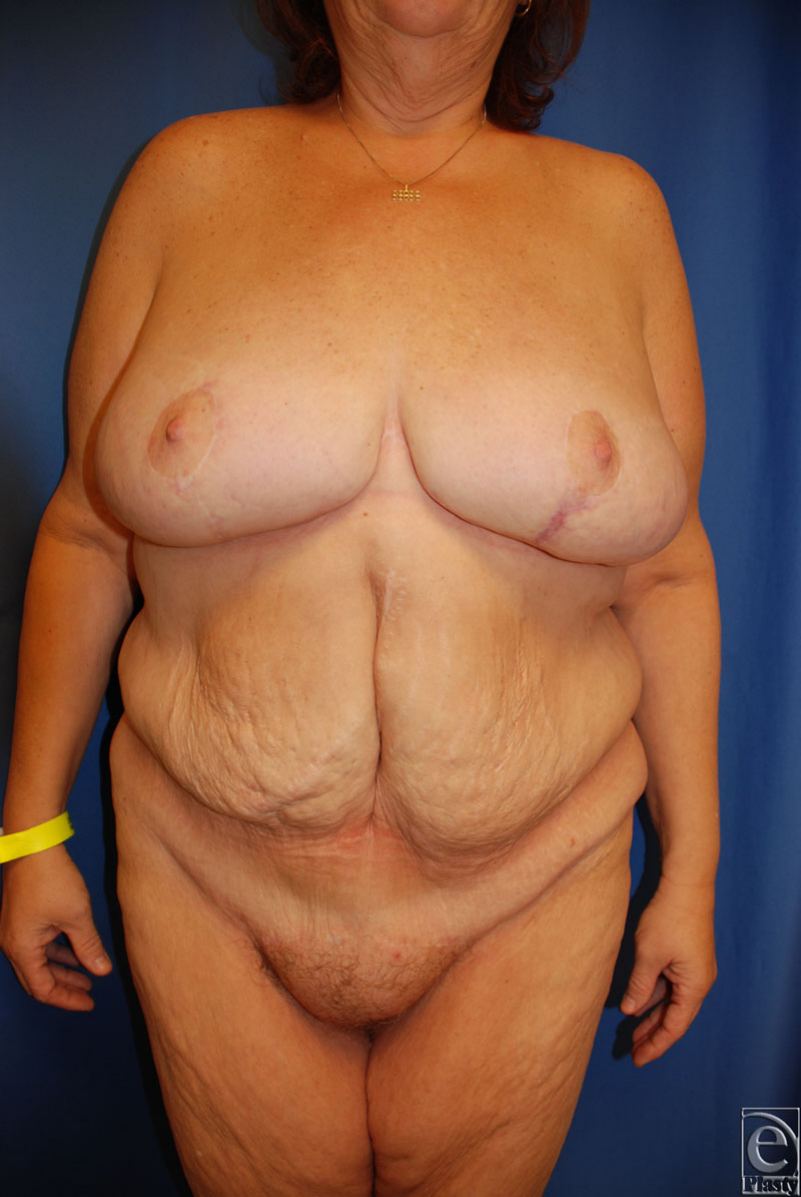

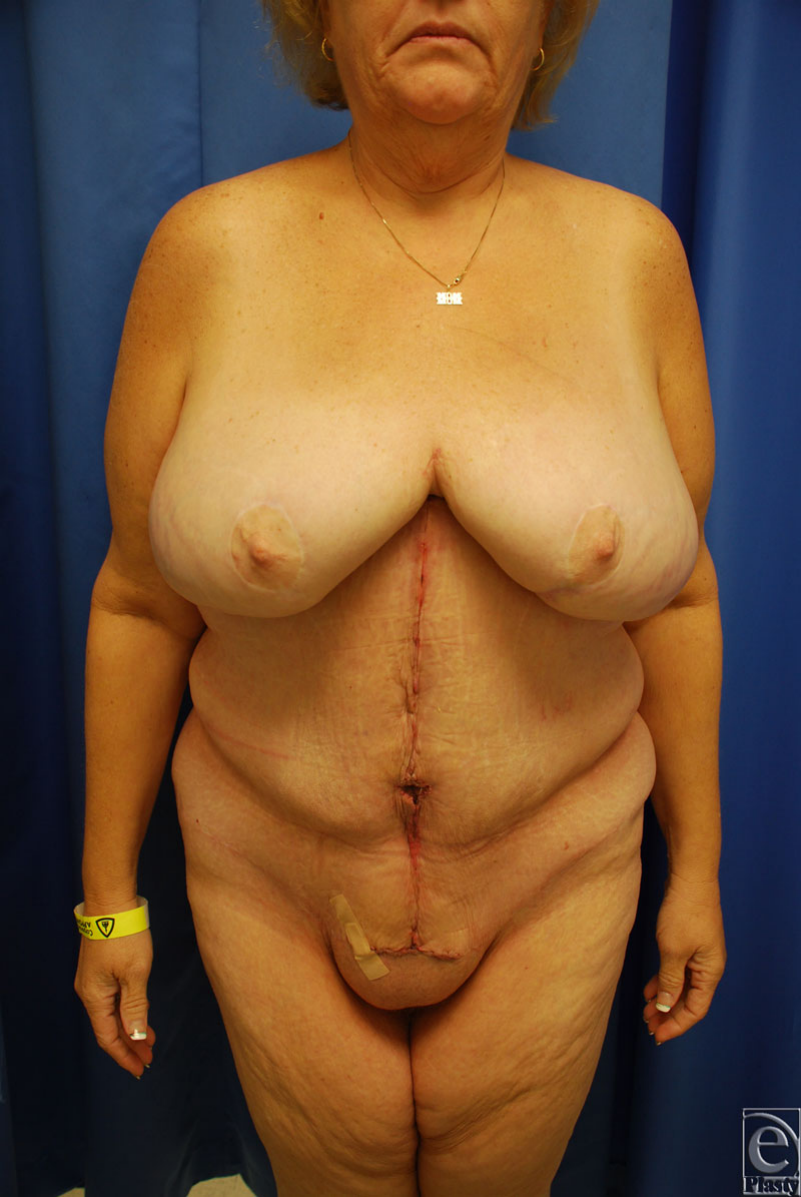
(*a*) This is a 49-year-old woman who had open gastric bypass surgery and a history of open cholecystectomy with right subcostal scar, which was relatively high. She lost 100 lbs to achieve a BMI of 27.6. Her medical history included continued diabetes mellitus after massive weight loss. (*b*) She underwent abdominoplasty panniculectomy with no undermining, with removal of 7 lbs of skin. (*c*) She returned for second-stage reverse abdominoplasty with revision of the midline scar.

**Figure 3:**
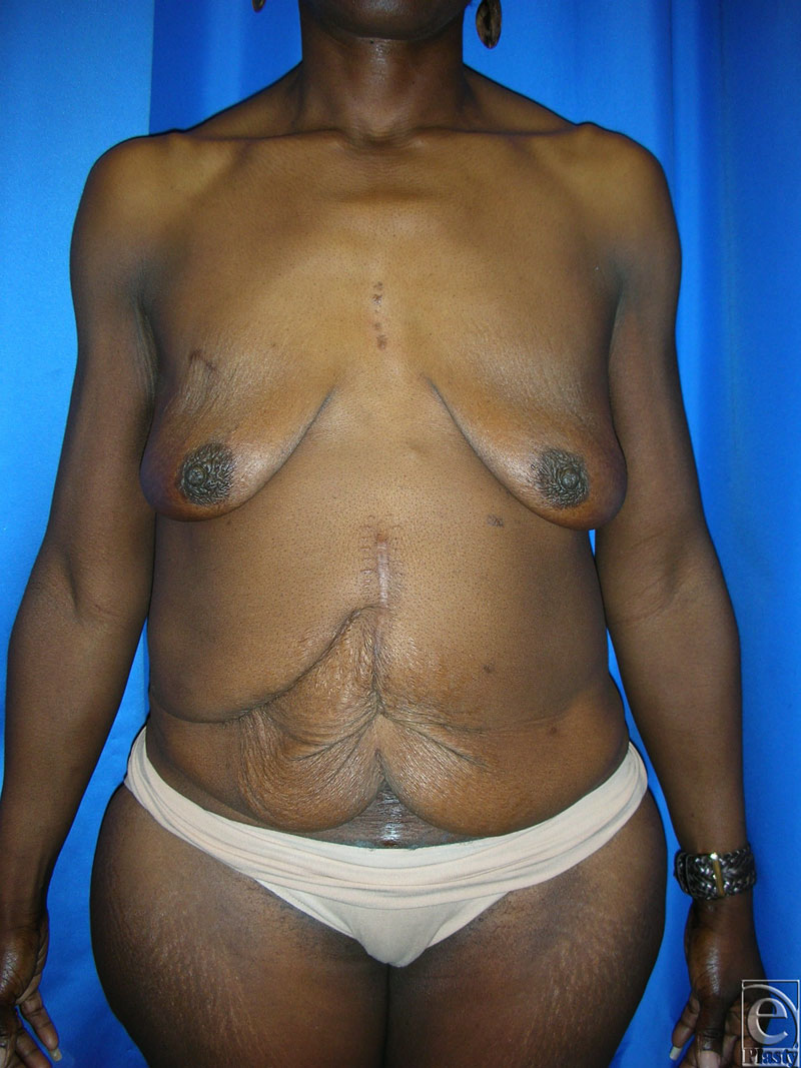

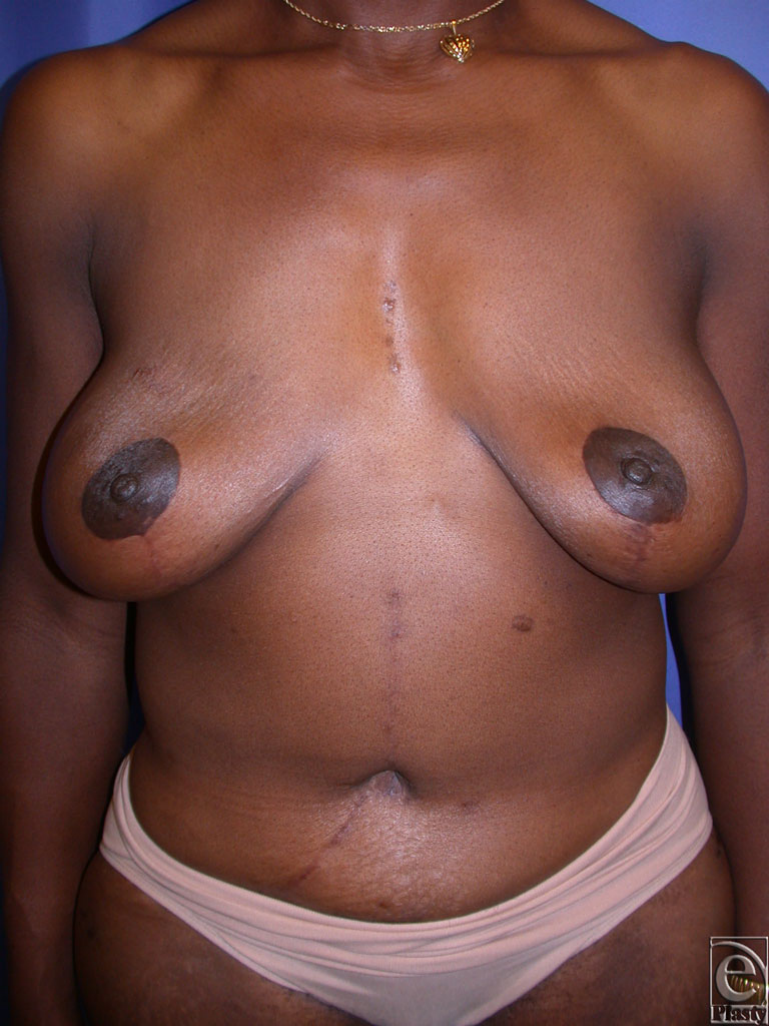
(*a*) This is a 50-year-old woman who had open gastric bypass surgery and a history of open cholecystectomy with right subcostal scar, which was relatively low. She lost 120 lbs to achieve a BMI of 25.6. (*b*) She underwent abdominoplasty surgery and abdominal wall plication with removal of all abdominal tissue below the subcostal scar. BMI indicates body mass index.

**Figure 4:**
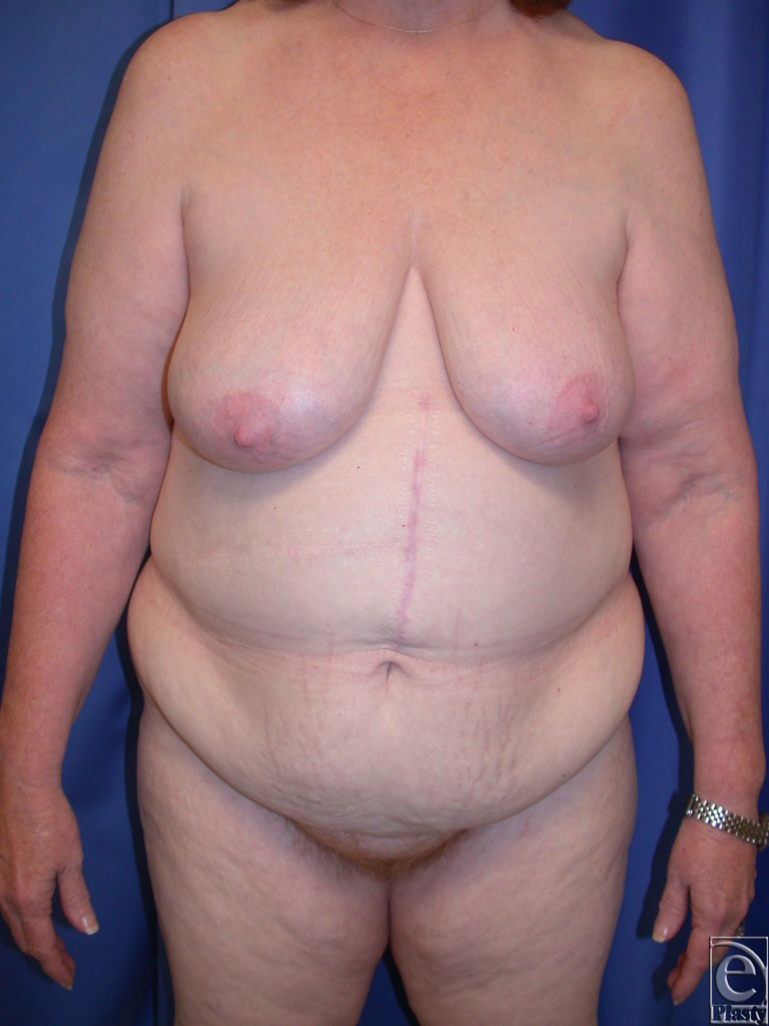

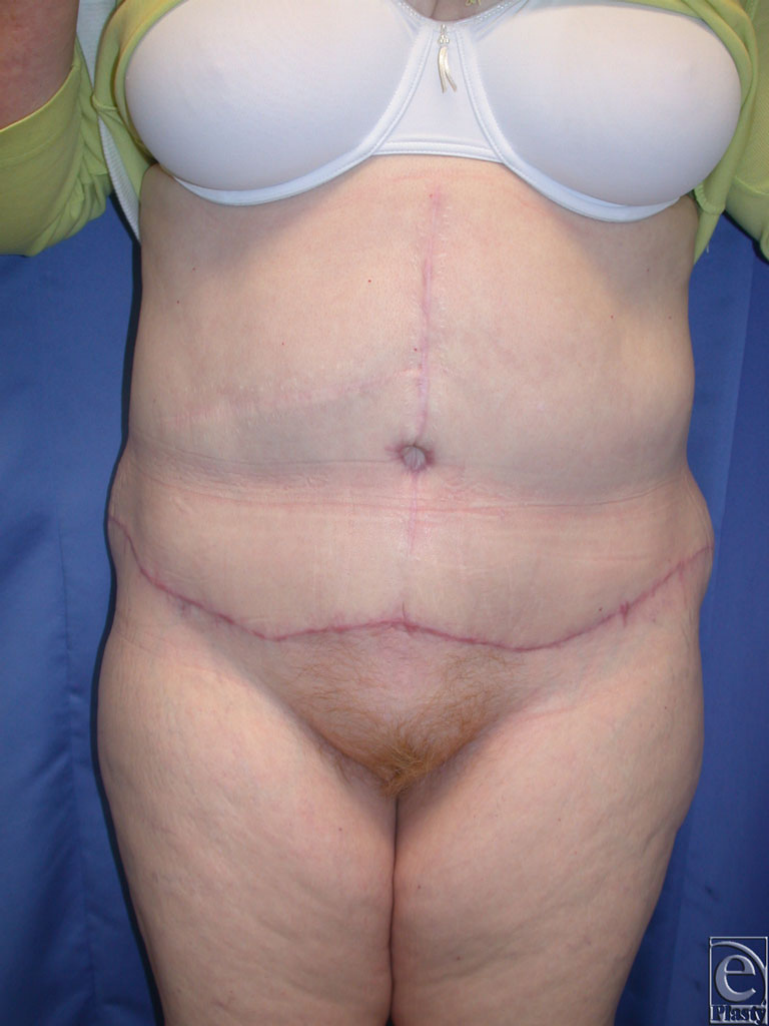
(*a*) This is a 57-year-old woman who had open gastric bypass surgery and a history of open cholecystectomy with right subcostal scar, which was relatively high. She lost 100 lbs to achieve a BMI of 29.1. (*b*) She had conservative panniculectomy without undermining and removal of 2.2 lbs of skin. BMI indicates body mass index.

We have not experienced significant detriment from subcostal scars because we designed our operative approach with the scar in mind, as supported by the studies cited in the breast reconstruction literature as well as several studies specifically investigating abdominal contouring in postbariatric patients.^11^^-^13 Our recommended strategy for abdominoplasty in patients with right subcostal scars depends on the patient's BMI and medical comorbidities known to impair wound healing, as well as the position of the subcostal scar. For patients presenting with elevated BMI and medical comorbidities, and especially if the subcostal scar is relatively high, we perform conservative panniculectomy, with minimal undermining in an effort to maximize the number of perforator vessels nourishing the skin (Fig 2). This approach emphasizes the alleviation of functional symptoms from overhanging skin and safety with minimal healing requirement. Later, staged removal of upper abdominal skin may be performed to improve contour. In healthy patients with BMI 30 or higher and a relatively low scar, we try to perform maximal removal of the skin below the scar, working the scar into the abdominal closure (Fig 3). For the patients who do not fall into these 2 broad categories, we individualize treatment taking into consideration medical comorbidities, BMI, and age: we will perform abdominoplasty in those patients we believe will do well, but with very careful undermining performed under the subcostal scar to optimize perforator vessel blood supply (Fig 4). Conversations take place preoperatively regarding the elevated risks of wound healing and seroma formation with subcostal scars. If there is any concern about viability of the abdominal skin, we err on the side of conservativism, with minimal undermining and panniculectomy of only the overhanging skin. Wound healing problems occurring subsequent to more aggressive undermining in the presence of abdominal scars often require long-term wound care and possible reoperation, both unpleasant possibilities for the surgeon and the patient.

Upper midline scars do not pose a problem with wound healing. In patients with upper midline scars, we do undermine the scars to allow the scar to migrate down to the pubis when performing abdominoplasty. We do not routinely excise these scars because of the concern about potential wound healing problems that might occur in the suprapubic region. Along these lines, we do not routinely perform fleur-de-lis excision in order to preserve maximal vascularity to the abdominal skin flap. With greater numbers of laparoscopic gastric bypass procedures being performed, we believe avoidance of a visible midline scar in abdominoplasty is preferable. For cases in which the upper midline scar results in contracture limiting the downward migration of the abdominal skin and closure of the skin to the pubis in the midline, we excise the scar through the dermis only, leaving subcutaneous fat intact.

As experience grows with body contouring for the MWL population, we can more clearly identify appropriate surgical procedures for those with specific medical and surgical history. Although we found here that subcostal scars do not directly impact surgical outcome, we do recommend conservative undermining and individualized treatment plans, particularly considering BMI.

## Figures and Tables

**Figure 1:**
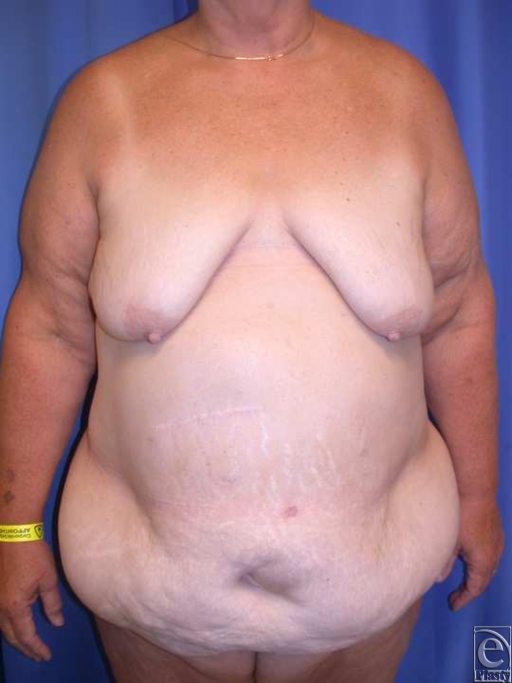

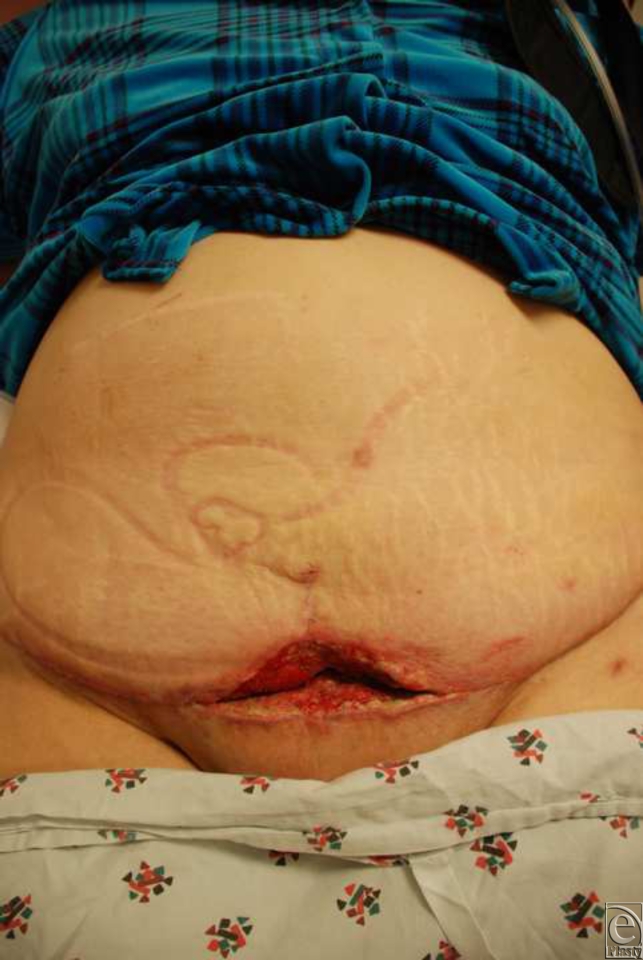
(*a*) This is a 61-year-old woman with history of laparoscopy gastric bypass surgery and 100 lb weight loss to BMI of 40. She underwent conservative panniculectomy in light of her morbid obesity and subcostal scar. (*b*) She sustained abdominal wall necrosis and was left with a large wound after surgical debridement. Vaccum-assited closure therapy optimized healing. BMI indicates body mass index.

**Table 1:** Demographic data*

Patient population, *N* = 420 (March 1998 through February 2008)	
Female:male = 372:48	
Mean age = 42 y (range = 20–72 y)	
Average BMI at body contouring = 33 (range = 19.5–88)	
**Patient group**	***N* (%)**
Open GBS (upper midline scar)	261 (62)
Laparoscopic GBS	80 (19)
Kocher scar	29 (7)
Cosmetic	79 (19)

*BMI indicates body mass index; GBS, gastric bypass surgery.

**Table 2:** χ^2^ Analysis: Any abdominal scar × any complication*

		Abdominal scar		
Any complication		0	1	Total
**0**	Frequency	134	146	340
	Row, %	47.86	52.14	100.00
	Column, %	75.71	60.83	67.15
**1**	Frequency	43	94	137
	Row, %	31.39	68.61	100.00
	Column, %	24.29	39.17	32.85
**Total**	Frequency	177	240	417
	Row, %	42.45	57.55	100.00
	Column, %	100.00	100.00	100.00

*Pearson χ^2^_1_ = 10.2149; *p* = 0.001.

**Table 3:** χ^2^ Analysis: Any abdominal scar × wound healing complication*

		Abdominal scar		
Wound complication		0	1	Total
**0**	Frequency	154	186	340
	Row, %	45.29	54.71	100.00
	Column, %	87.50	77.50	81.73
**1**	Frequency	22	54	76
	Row, %	28.95	71.05	100.00
	Column, %	12.50	22.50	18.27
**Total**	Frequency	176	240	416
	Row, %	42.31	57.69	100.00
	Column, %	100.00	100.00	100.00

*Pearson χ^2^_1_ = 6.8002; *p* = 0.009.

**Table 4:** χ^2^ Analysis: Kocher scar × wound healing complication

		Kocher scar		
Wound complication		0	1	Total
**0**	Frequency	317	18	335
	Row, %	94.63	5.37	100.00
	Column, %	83.64	62.07	82.11
**1**	Frequency	62	11	73
	Row, %	84.93	15.07	100.00
	Column, %	16.36	37.93	17.89
**Total**	Frequency	379	29	408
	Row, %	92.89	7.11	100.00
	Column, %	100.00	100.00	100.00

*Pearson χ^2^_1_ = 8.5333; *p* = 0.003.

**Table 5:** Multiple logistic regression statistical analysis: Any complication*

	Odds ratio	*P* > |z|	95% CI
Kocher scar	0.9003291	.815	0.3737069	2.169059
Age	1.018154	.137	0.9942878	1.042592
Female gender	0.3955095	**.006**	0.2029569	0.7707437
Diabetes	1.472378	.272	0.7380568	2.937302
Cardiac disease	0.8647392	.775	0.3193484	2.341562
Tobacco use	1.224361	.540	0.6412066	2.337873
BMI at contour	1.055066	**.000**	1.026275	1.084665

*BMI indicates body mass index.

**Table 6:** Multiple logistic regression statistical analysis: Wound complication*

	Odds ratio	*P* > |z|	95% CI	
Kocher scar	2.178394	.088	0.8897298	5.333532
Age	1.020947	.166	0.991409	1.051366
Female gender	0.6055355	.188	0.2869131	1.277994
Diabetes	1.653966	.183	0.7892762	3.465964
Cardiac disease	1.353437	.570	0.4764891	3.84435
Tobacco use	1.591883	.233	0.7417811	3.416224
BMI at contour	1.0546	**.001**	1.023207	1.086956

*BMI indicates body mass index.

## References

[1] American Society of Plastic Surgeons 2008 report of the 2007 statistics: National Clearinghouse of Plastic Surgery Statistics. http://www.plasticsurgery.org/d.xml?comp=x3309.

[2] Parrett BM, Caterson SA, Tobias AM, Lee BT (2008). DIEP flaps in women with abdominal scars: are complication rates affected?. Plast Reconstr Surg.

[3] Losken A, Carlson GW, Jones GE, Culbertson JH, Schoemann M, Bostwick J (2002). Importance of right subcostal incisions in patients undergoing TRAM flap breast reconstruction. Ann Plast Surg.

[4] Schoeller T, Huemer GM, Kolehmainen M, Otto-Schoeller A, Wechselberger G (2004). Management of subcostal scars during DIEP-flap raising. Br J Plast Surg.

[5] Shermak MA, Chang D, Magnuson TH, Schweitzer MA (2006). An outcomes analysis of patients undergoing body contouring surgery after massive weight loss. Plast Reconstr Surg.

[6] Shermak MA, Chang DC, Heller J (2007). Factors impacting thromboembolism after bariatric body contouring surgery. Plast Reconstr Surg.

[7] Nemerofsky RB, Oliak DA, Capella JF (2006). Body lift: an account of 200 consecutive cases in the massive weight loss patient. Plast Reconstr Surg.

[8] Au K, Hazard SW, Dyer AM, Boustred AM, Mackay DR, Miraliakbari R (2008). Correlation of complications of body contouring surgery with increasing body mass index. Aesthet Surg J.

[9] Chang DW, Wang B, Robb GL (2000). Effect of obesity on flap and donor site complications in free transverse rectus abdominis myocutaneous flap breast reconstruction. Plast Reconstr Surg.

[10] Spear SL, Ducic I, Cuoco F, Taylor N (2007). Effect of obesity on flap and donor-site complications in pedicled TRAM flap breast reconstruction. Plast Reconstr Surg.

[11] Rieger UM, Aschwanden M, Schmid D, Kalbermatten DF, Pierer G, Haug M (2007). Perforator-sparing abdominoplasty technique in the presence of bilateral subcostal scars after gastric bypass. Obes Surg.

[12] Rieger UM, Erba P, Kalbermatten DF, Schaefer DJ, Pierer G, Haug M (2008). An individualized approach to abdominoplasty in the presence of bilateral subcostal scars after open gastric bypass. Obes Surg.

[13] Kolker AR (2008). Improving esthetics and safety in abdominoplasty with broad lateral subcostal perforator preservation and contouring with liposuction. Ann Plast Surg.

